# Delayed diagnosis of duodenal duplication cyst in a child with recurrent pancreatitis operated by Whipple procedure: a case report

**DOI:** 10.1097/MS9.0000000000000792

**Published:** 2023-05-09

**Authors:** Abdalhakim R. M. Shubietah, Soud M. S. Deek, Zaid Zakaria, Fares Saleh, Abdulmotti Abdulkareemsrour, Mohamad Naser, Yazan M.S. Dibas, Mohammed S.M. Jabri, Haytham M. A. AbuMohsen

**Affiliations:** a Palestinian Ministry of Health, Darwish Nazzal Government Hospital, Qalqilya; b Palestinian Ministry of Health; c Department of Surgery; d Department of Emergency Medicine, Rafidia Government Surgical Hospital; e Faculty of Medicine and Health Sciences, An-Najah National University, Nablus; f Department of Emergency Medicine, Palestinian Medical Complex, Ramallah; g Palestinian Ministry of Health, Tubas Government Hospital, Tubas, Palestine

**Keywords:** acute recurrent pancreatitis, case report, duodenal duplication cyst, pancreatitis, Whipple

## Abstract

Children with acute pancreatitis may experience abdominal pain; the causes of which include anatomical abnormalities and medications. The rare anatomical etiology of duodenal duplication cyst is typically asymptomatic and discovered incidentally. The authors present a unique case of a Whipple-operated duodenal duplication cyst.

**Case presentation:**

The authors present the case of a 12-year-old Arab male who was admitted to our hospital with worsening, severe epigastric pain, stabbing in nature, and radiating to the back, suggestive of acute pancreatitis. Serum lipase levels were significantly elevated. The patient received appropriate care. His medical history is notable for multiple bouts of pancreatitis in the last 18 months. Previous investigations at other hospitals were mostly unrevealing. A more extensive workup was performed, revealing a duodenal cystic structure. This led to the diagnosis of a duodenal duplication cyst. The Whipple procedure was decided upon due to his recurrent pancreatitis, which caused fibrotic adhesions and anatomic region distortion. The patient underwent surgery and recovered uneventfully.

**Discussion:**

Acute pancreatitis in children is frequent and can be caused by unrecognized duodenal duplication cysts. When symptomatic, the majority present with vague abdominal pain. Despite using ultrasound as an adjunctive for diagnosis, the cyst might not become apparent until the condition has progressed to an advanced stage. A delayed diagnosis may result in complications necessitating risky surgeries.

**Conclusion:**

Children with recurrent pancreatitis should be evaluated for a duodenal duplication cyst. Early use of computed tomography scans may be necessary to identify the cause and spare the patient from risky procedures like Whipple surgery.

## Introduction

Highlights

- Duodenal duplication cyst is a challenging diagnosis, often incidentally discovered in children below 2 years of age.
- Ultrasound may not always detect duodenal duplication cysts in children, and a computed tomography scan, although invasive, may be necessary for an accurate diagnosis.
- Early use of CT scans may be advisable in suspected cases of duodenal duplication cysts to avoid a delayed diagnosis.
- Delayed diagnosis of duodenal duplication cysts can lead to potentially fatal consequences and impact the quality of life.
- An endoscopic approach is the usual treatment for duodenal duplication cysts, but the Whipple procedure may be required in certain select cases.

Acute abdominal pain is one of the most common complaints in children, and it presents a diagnostic challenge due to various underlying causes^1^. Acute pancreatitis as a cause of abdominal pain has become a common general pediatric condition in the last two decades, with an increasing incidence^2^. The causes vary but are frequently drugs, infections, trauma, or anatomic anomalies^3^. According to the INSPPIRE Group (International Study Group of Pediatric Pancreatitis: In Search for a Cure), acute pancreatitis is defined as reversible inflammation of the pancreatic parenchyma when two of the three following criteria are met: abdominal pain compatible with acute pancreatitis, a serum amylase or lipase value three times the upper limit of normal, and imaging findings consistent with acute pancreatitis. Acute recurrent pancreatitis is defined as two distinct episodes of acute pancreatitis (each as defined above), with a return to baseline (either complete resolution of pain (one-month pain-free interval episodes) or complete normalization of serum pancreatic enzyme levels) in between^4^.

A duodenal duplication cyst is a severe but uncommon cause of acute pancreatitis. It makes up just 4% of all gastrointestinal tract duplications and is thought to occur less frequently than once per 100 000 live births^5^. Most duodenal duplication cysts are discovered in the first two decades of life and are uncommon, usually asymptomatic, and found incidentally^6–8^. This report describes a rare case of a 12-year-old Arab male with recurrent bouts of pancreatitis who received a delayed diagnosis of a duodenal duplication cyst and was operated on by the Whipple procedure. This manuscript complies with the Surgical CAse REport (SCARE) criteria^9^.

## Presentation of the case

A 12-year-old male of Arab descent was admitted to our hospital after presenting to the emergency room with worsening, severe, constant epigastric pain of a 1 h duration that was stabbing in nature and radiating to the back. The pain was unbearable and accompanied by nausea and twice vomit. His medical history was significant for multiple bouts of pancreatitis over the past 18 months, which had been managed at other hospitals. Between his first attack and his current presentation, it was noted that his BMI had dropped from 29 to 25 kg/m^2^.

Previous investigations during the first few attacks, including ultrasound (US) and blood tests, did not yield conclusive results on a specific underlying cause for pancreatitis. However, during a previous attack, a computed tomography (CT) scan without contrast was performed, which revealed the presence of a cystic structure, as reported by the family. However, no formal report was found, and no treatment was recommended for the cyst at that time. The patient had also experienced intermittent, vague abdominal pain for the past year, which had been attributed to indigestion. His surgical history was unremarkable, and no significant drug or family history was reported. Further questioning revealed no history of recent illnesses, substance use, abdominal trauma, or scorpion bite.

Upon physical examination, the patient was found to be tachypneic with a temperature of 38.3°C, a heart rate of 100, a blood pressure of 105/67, and an oxygen saturation of 96% on room air. Clinical examination of the abdomen revealed tenderness over the epigastric area with voluntary guarding but was otherwise negative. A complete blood count, amylase, lipase, and other relevant lab tests were obtained, and results are shown in Table 1. Based on the patient’s medical history, physical examination findings, and significantly elevated serum lipase level that was three times the upper limit of normal, a diagnosis of acute pancreatitis was made.

**Table 1:** Laboratory test results.

Labs	Result	Normal reference
WBC	8000	4.5–11.0×10^9^/l
Neutrophils %	76	40–60%
Serum Amylase	270	28–100 U/L
Serum Lipase	520	0–160 U/L
AST	21	0–50 U/L
ALT	17.7	0–41 U/L
Serum Calcium	9.93	8.4–10.2 mg/dl
BUN	10.2	7–30 mg/dl
Total Bilirubin	0.5	0–1.2 mg/dl
ALP	144	40–129 IU/L

As part of routine tests, alpha-fetoprotein, cancer antigen 19-9 (CA 19-9), and carcinoembryonic antigen levels were obtained, and results were within the normal range. The patient was started on total parenteral nutrition, made nil per os, and received intravenous fluids and omeprazole as part of routine management for acute pancreatitis. Further evaluation and management, including consideration of the previously identified cystic structure, were planned to determine the underlying cause of recurrent pancreatitis in this patient.

**Figure 1:**
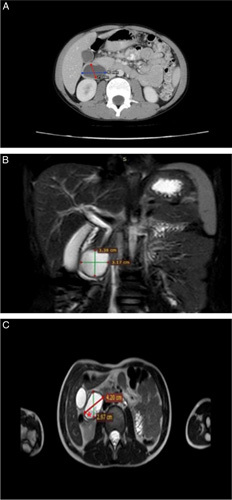
(A) Computed tomography scan with oral and IV contrast shows a 2.3 cm anteroposterior ×4 cm transverse well-defined cystic lesion posteroinferior to the pancreatic head. The cyst is intimately related to the second part of the duodenum but not contrasted with oral contrast. (B and C) MRCP shows a well-defined fluid-filled cystic structure between the head of the pancreas and the second/third duodenal junction measuring about 4.2 cm transverse ×3.38 cm craniocaudal ×2.97 cm anteroposterior in maximal dimensions. The cyst shows dependent debris without definite air bubbles (red asterisk) with a mass effect on the duodenum and head of the pancreas, resulting in minimal dilatation of the common bile duct.

A 4×3 cm cystic structure with a well-defined wall and internal debris seen posteroinferior to the pancreatic head and medial to the concavity of the duodenum was detected by bedside abdominal US. To assist in locating the cyst, a CT scan with and without oral and intravenous fluids contrast was ordered. The pancreatic body and tail appeared bulky with peripancreatic free fluid; however, it showed homogenous enhancement. As shown in Figure 1A, there is an about 2.3×4 cm well-defined cystic lesion posteroinferior to the pancreatic head.

Although CT with oral contrast was done, a barium swallow was ordered by another team member. This unnecessarily exposed the patient to more radiation. However, it unsurprisingly showed a filling defect in the medial aspect of the second part of the duodenum forming an acute angle with the duodenal wall. The cyst was then identified using magnetic resonance cholangiopancreatography (MRCP), which revealed a well-defined fluid-filled cystic structure with maximal dimensions of approximately 4.2×3.38×2.97 cm between the head of the pancreas and the second/third duodenal junction as shown in Figure 1B and C.

Previous investigations suggested that the most likely diagnosis was a duodenal duplication cyst or choledochal cyst (choledochocele). However, recent MRCP results demonstrated that the cyst and common bile duct are not connected, confirming that the cyst is indeed a duodenal duplication cyst.

Considering the recurrent nature of the patient’s pancreatitis, it was initially hypothesized that fibrotic adhesions and anatomical distortion might necessitate the Whipple procedure. The surgical team discussed various surgical and endoscopic options with the family, and together they agreed that converting to the Whipple procedure, instead of simply removing the cyst, may be the ultimate course of action. The patient was admitted to the hospital for elective surgery, and during the procedure, the initial assumptions were confirmed, leading to the decision to proceed with the Whipple procedure.

The surgical procedure involved several steps. Firstly, the duodenum was divided and mobilized, followed by the opening of the lesser sac and the division of the inferior border of the duodenum. The greater curvature of the stomach was then mobilized, and the distal part of the stomach was resected using blue GIA 80. A jejunum loop was also resected using blue GIA 60. The superior mesenteric artery was identified, and the pancreas was resected and disconnected from the retroperitoneum using 2/0 silk sutures and then removed en bloc.

Next, the pancreas was anastomosed with the posterior wall of the stomach using an umbrella suture. End-to-end hepaticojejunostomy was performed using a continuous 5/0 prolene suture, and the distal jejunal loop was anastomosed end-to-side with the stomach using 4/0 PDS sutures. Entero-enterostomy was performed 80 cm distally to the biliary anastomosis using 4/0 PDS sutures. Two JP-10 drains were left in the abdomen adjacent to the anastomoses to facilitate postoperative drainage.

The abdomen was closed using a two-layer technique, with the inner layer closed using continuous 3/0 vicryl sutures, and the external layer closed using interrupted 2/0 vicryl sutures. The skin was closed with a stapler.

**Figure 2:**
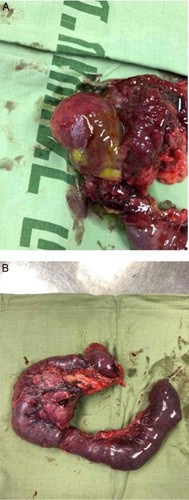
(A) Resected duodenum with a bulge (representing duodenal duplication cyst). The head of the pancreas is shown on the right. (B) Shows the resected duodenum with the head of the pancreas.

**Figure 3:**
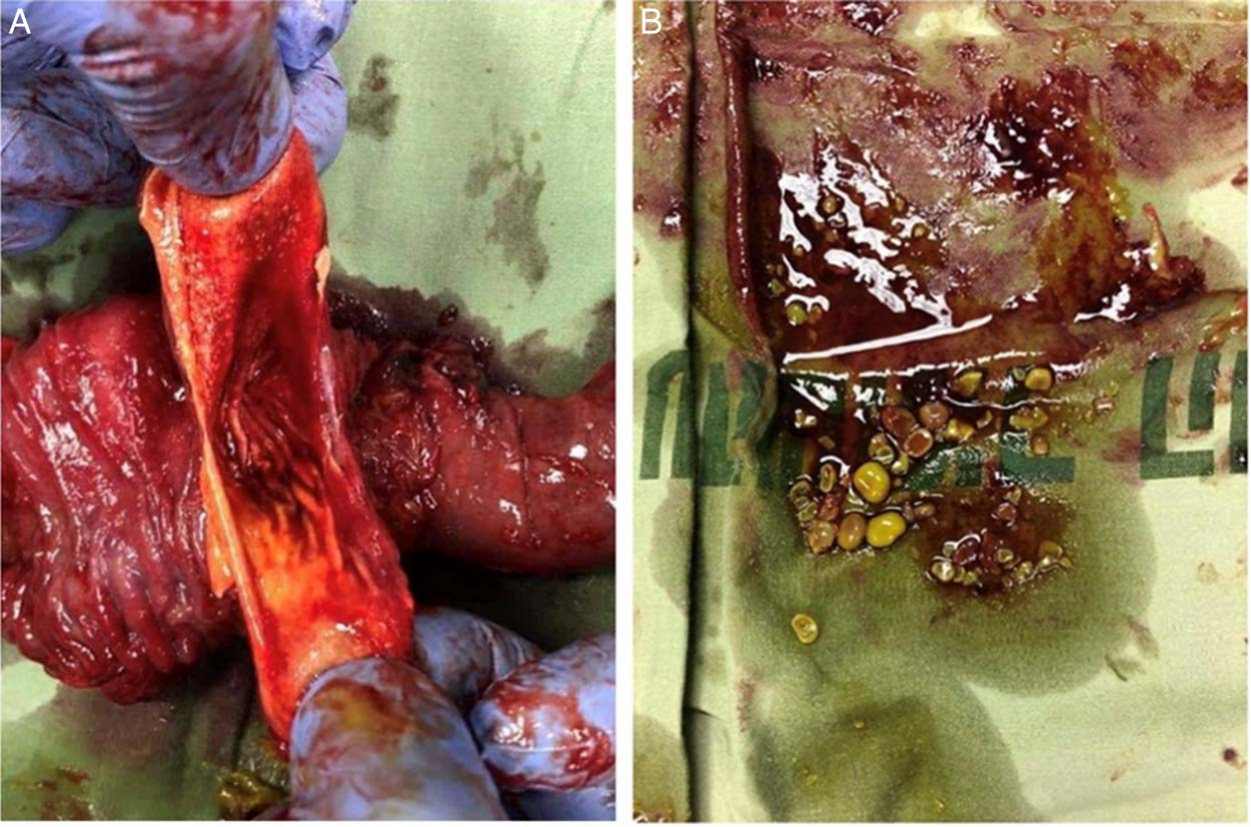
(A). The duodenal duplication cyst is shown open. (B). Stones are retrieved from the cyst when drained of its contents.

In summary, the patient underwent the Whipple procedure with distal gastrectomy and duodenectomy with ROUX-EN-Y anastomosis of the stomach to the jejunum (Figs. 2 and 3). The procedure was uneventful, and the cyst identified as arising from the papilla was sent to the pathology lab for evaluation, with results showing no features indicative of malignancy.

The patient was transferred to the pediatric ICU for monitoring after the procedure. He was initiated on a total parenteral nutrition formula consisting of 3% amino acid and 3% glycerin injection with electrolytes, as well as receiving ranitidine, somatostatin, and ceftriaxone. After two days, the patient was discharged from the ICU and transferred to the pediatric surgical ward. During his postoperative period, the patient’s recovery was uneventful, with clinical improvement noted in both the physical examination and laboratory tests. The patient was able to tolerate oral feeding, had normal bowel movements, and passed gas. The plan was to discharge the patient on postoperative day seven. Until now, follow-up evaluations, including assessments of surgical site healing, recovery of bowel function, and resolution of postoperative symptoms, were unremarkable. Physical examinations, laboratory tests, and bedside transabdominal US did not reveal any concerning findings.

## Discussion

We report an unusual case of a duodenal duplication cyst in a pediatric patient, located in the second part of the duodenum, that was identified after 18 months of recurrent pancreatitis.

Acute pancreatitis is a condition that occurs frequently and presents with a wide range of symptoms. In extreme cases, it can be fatal^10^. Duodenal duplication cysts, which are congenital disorders associated with pancreatitis, are usually diagnosed in the first two years of life^6^. However, our case was unique as it was diagnosed at the age of 12, after experiencing multiple episodes of pancreatitis. While imaging is not necessary for diagnosing acute pancreatitis if the other 2 criteria are met, transabdominal US is often used to rule out any obstructive causes^4^. CT imaging is usually reserved for more severe cases where there is no clinical improvement or worsening^10^. In our case, despite undergoing imaging and blood tests, it was only 18 months after the initial symptoms began that an abdominal US revealed a cyst near the pancreas. This delay in detection could be attributed to bowel gas interference, small cyst size, or challenges in interpreting results due to the patient’s overweight condition. The patient had experienced intermittent, vague abdominal pain for a year before the diagnosis. This aligns with reports that suggest the most common symptoms of duodenal duplication cysts are nonspecific, such as vague abdominal pain with or without nausea and vomiting^11^, which can be easily mistaken for other causes like indigestion.

Limited research exists on acute recurrent pancreatitis in children, with an uncertain understanding of its epidemiology and natural history. Furthermore, there is a lack of evidence-based guidelines for diagnosis, prognosis, and treatment in pediatric cases, leading to reliance on adult-derived guidelines by pediatric specialists. This poses challenges as the causes of pancreatitis in children differ significantly from those in adults^4^.

Due to the variability in presenting symptoms and challenges in diagnostic imaging, duodenal duplication cysts pose a significant risk of morbidity and mortality if not diagnosed and treated promptly^12^. Diagnostic tools such as abdominal US, CT scan, MRI, and MRCP have been commonly employed in reported cases, based on clinical suspicion. Additionally, upper gastrointestinal series and endoscopic retrograde cholangiopancreatography have been utilized for diagnostic purposes. Endoscopic US has also been employed in certain cases as a diagnostic modality^5^.

The preferred treatment approach for duodenal duplication cysts is complete excision. However, less invasive methods such as partial resection or internal derivation have also been suggested as alternative approaches. Another successful surgical technique, even when performed endoscopically, is marsupialization^13^. While endoscopic excision is commonly used in pediatric cases^5^, our patient underwent a Whipple procedure due to intraoperative findings of fibrotic adhesions and distortion of the anatomic region resulting from recurrent pancreatitis. This approach is similar to a reported case where a 23-year-old patient underwent a Whipple procedure due to the firm adherence of a necrotic and inflammatory mass to the duodenum and pancreatic head^14^. However, the patient in our case was 12-year-old when the surgery took place. Deciding whether a Whipple procedure should be the first surgical option for pediatric patients with a benign disease like a duodenal duplication cyst requires extensive deliberation. This approach is uncommon, as supported by a previous case series study where only one out of 11 cases underwent the Whipple procedure^15^.

Although it is generally discouraged to utilize silk sutures for closing abdominal incisions^16^, in low-resource settings like Palestine, where healthcare resources may be scarce and substandard, silk sutures are still commonly employed^17,18^. Additionally, a national survey conducted by Brunner *et al*.^19^ found that unlike the prolene sutures used in our case for end-to-end hepaticojejunostomy, all hospitals surveyed uniformly used monofilament absorbable sutures. This practice could also be attributed to resource limitations in our facilities.

The cyst needs to be surgically removed as it may cause severe symptoms and there’s a potential risk of malignant transformation. Long-term follow-up is necessary to monitor for any potential malignant changes at the site of the duplication^20^. In our patient, the resected cyst originated from the papilla, and the biopsy results confirmed it was benign.

## Conclusion

Regardless of the patient’s age, it is crucial to consider the possibility of a duodenal duplication cyst when there is a history of recurrent pancreatitis. Although US is typically the initial diagnostic imaging modality in children, we believe that earlier utilization of CT scans and a comprehensive workup, such as MRCP, may be warranted when US findings are inconclusive, blood tests are negative, and medical history does not suggest an underlying cause for recurrent pancreatitis. Further research is needed to determine the sensitivity of abdominal US in detecting anatomical causes of pancreatitis and to establish a distinct diagnostic approach for recurrent pancreatitis in children compared to adults. Early recognition and management of duodenal duplication cysts could potentially reduce the need for invasive surgeries like the Whipple procedure. Despite the rarity of this type of surgery in children, it should be considered in cases where significant adhesions and distortion of the anatomical region are present, as alternative treatments may not effectively address the patient’s condition.

## Ethical approval

Ethical approval is exempt/waived at our institution.

## Consent

The patient’s parents provided written informed consent to publish this case report and accompanying images. A copy of the written consent is available for review by the Editor-in-Chief of this journal upon request.

## Sources of funding

NA.

## Author contribution statement

S.D., A.S.: study concept and design; F.S., A.A., M.N.: data collection; S.D., H.A.M., A.S.: data analysis and interpretation; S.D., A.S., F.S., A.A., H.A.M., M.N.: writing the paper; Z.Z., Y.D., M.J., A.S.: writing - review and editing.

## Conflicts of interest disclosure

NA.

## Research registration unique identifying number (UIN)

NA.

## Guarantor

Abdalhakim Shubietah.

## Data availability statement

Available upon the Editor-in-Chief request.

## Provenance and peer review

Not commissioned, externally peer-reviewed.
